# Malignant Nodular Hidradenoma of the Left Knee: A Malignant Mystery With an Overview of Literature

**DOI:** 10.7759/cureus.27454

**Published:** 2022-07-29

**Authors:** Narendhar Gokulanathan, Pandjatcharam Jagadesan, Kalaranjani M, Rajesh Nachiappa Ganesh

**Affiliations:** 1 Radiation Oncology, Jawaharlal Institute of Postgraduate Medical Education and Research, Puducherry, IND; 2 Pathology, Jawaharlal Institute of Postgraduate Medical Education and Research, Puducherry, IND

**Keywords:** ­skin cancer, geriatric oncology, skin adnexal tumors, radiotherapy (rt), nodular hidradenoma

## Abstract

Malignant nodular hidradenoma is a rare eccrine sweat gland neoplasm characterized by recurrence, metastasis, and a locally aggressive course. In our case report, a 74-year-old man presented with a seemingly benign swelling which was persistent for the last 30 years, which was excised at an outside institute. Since the patient presented to the hospital during the peak of the pandemic, considering the age of the patient, the pandemic situation, the logistics of radiotherapy during such a situation, preserving the knee joint function in view of close proximity of the tumour to the joint, it was decided to keep the patient on follow up and continue expectant management. After a follow-up period of 24 months, no locoregional recurrence or metastasis has been observed. The patient is on annual follow-up with clinical examination and PET-CECT imaging.

## Introduction

Malignant nodular hidradenoma is a rare eccrine sweat gland neoplasm notable for recurrence, metastasis, and a locally aggressive course, and has an incidence of 0.001% [1]. Usually involving the head and neck region and the anterior truncal surface, eccrine tumours of the extremities are extremely rare. The treatment of choice is wide local excision and the role of adjuvant therapy is controversial [2]. Here we report a case of malignant nodular hidradenoma in the lower leg of an elderly gentleman, aged 74, post-resection.

## Case presentation

History and clinical assessment

Our patient initially presented with a complaint of swelling over the lateral aspect of the left knee. The swelling was persistent over the area for 30 years previously and was not associated with any symptoms till recently. The swelling started becoming painful over the last two months. The pain was dull aching in type with no temporal variation, had no aggravating factors, and was relieved on taking analgesics. It was not associated with trauma, ulcers, or pre-existing scars. The patient did not report any discharge from the swelling, nor any redness. No secondary changes like ulceration, fungating, and softening were observed. There was no history of similar lumps in the body, and it was not associated with fever. He did not report any bleeding or suppuration from the lesion.

Initially, the swelling was 50x40 mm in size, with a nodular surface. Patient-reported mild tenderness on palpation. The swelling was firm in consistency. No fixity to local structures was observed. No distal neurovascular deficits were observed. No ulceration, erythema, or tenderness was present over the swelling. No discharge, bleeding, nor locoregional lymph node enlargement was present. No systemic symptoms were present. Differential diagnoses were fibroma, synovial or meniscal cyst, lipoma, and sarcoma.

Surgical excision of the lesion was planned at an outside institute and the patient was referred to our institute for subsequent management.

Post-operative histopathological examination of excision biopsy done at another institute

A nodular swelling measuring 30x35x25 mm is observed, its cut surface is grey white and lobulated with cystic spaces. Sections show skin and dermis and lobulated tumour composed of anastomosing cords of cuboidal cells, loose mesenchymal stroma, and cystic spaces lined by cuboidal cells and filled with proteinaceous material. The focal area shows sheets of large cells with large vesicular nuclei and prominent nucleoli. The superior, inferior, medial, lateral, and deep resected margins are free of tumour, with the closest margin being the deep resected margin, which measured 19mm. Overall features were suggestive of a malignant nodular hidradenoma.

**Figure 1:**
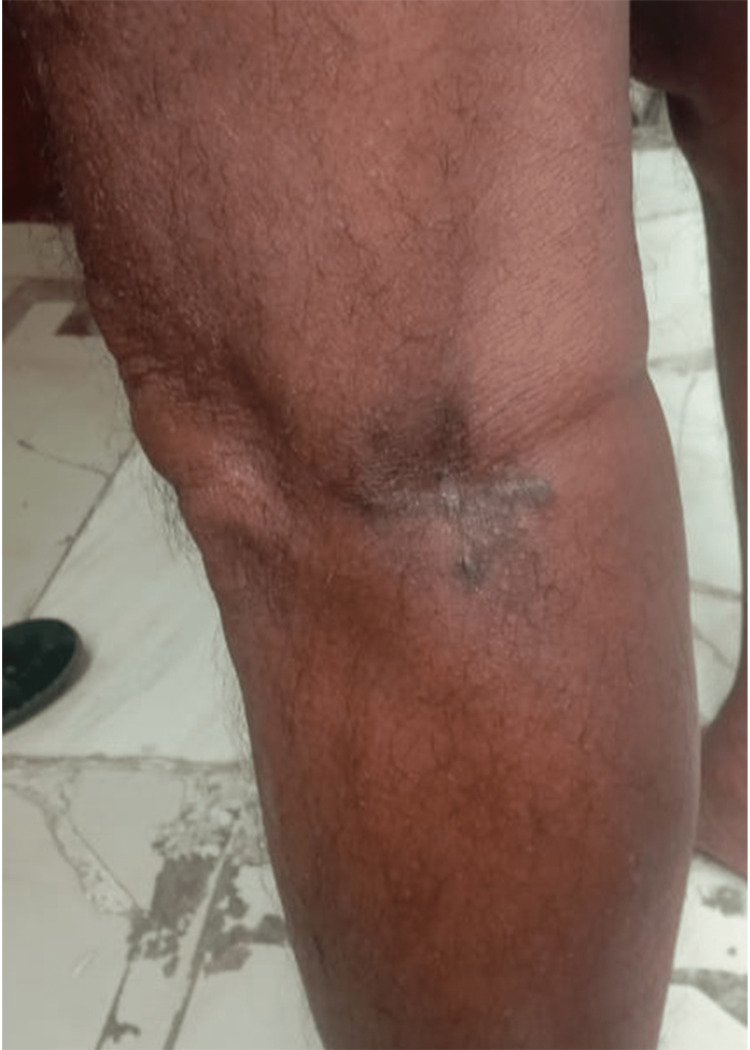
Post-operative image of the site of lesion

On examination, a 66 mm linear scar was present over the posterolateral aspect of the left knee with no residual swelling. The skin on and around the scar is thickened. There was no tenderness on palpation. A picture of the postoperative site is attached in Figure 1.

MRI of the left knee

A T1 and T2 hypointense irregular scar involving skin and the subcutaneous plane of the posterolateral aspect of the knee is seen. There is no extension into the deep fascia. On post-contrast scans, mild enhancement is seen. Mild effusion is also seen. Periarticular soft tissue planes appear normal. No suspicious features were noted. As a result, re-excision was deferred.

Histopathological slide review at the institute

**Figure 2:**
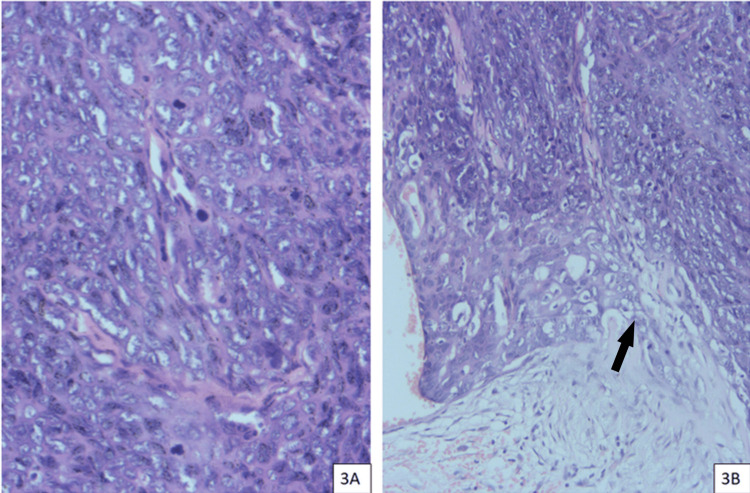
Section shows highly cellular solid focus with high mitotic activity. Hematoxylin and eosin stain, x 400

The histopathological slides of the operated lesion were referred for further analysis to our institute. This section shows skin with an epidermis that is unremarkable. The deep dermis shows a tumour with cells arranged in solid sheets, nests, and anastomosing cords with intervening cystic spaces filled with eosinophilic material. Cells show moderate pleomorphism with a vesicular nucleus and prominent nucleoli with mitotic activity. Tumour cells seem to invade as tiny nests into the subcutaneous adipose tissue. Features are those of a malignant adnexal tumour, consistent with malignant nodular hidradenoma. The tumour was positive for cytokeratin 5 and 6, EGFR, and EMA. The MIB-1 labelling index was 28%. Histopathological pictures are available for review in Figure 2. It was a fairly straightforward diagnosis and no other differentials were available for the above histopathological and immunohistochemical characteristics.

Follow-up protocol and further management

Treatment modalities for malignant nodular hidradenomas are under-researched and few in literature. Considering the age of the patient, the close proximity of the post-operative tumour cavity to the knee joint, and the pandemic situation, expectant management seemed a prudent course of action to follow. Our patient was put on close monitoring and is on regular follow-up as a part of expectant management.

As a part of expectant management, the patient underwent a PET-CT scan in June 2022 and there was no FDG uptake in the primary site or any distant sites. The patient is asymptomatic, has not reported any new swellings, and is on regular follow-up under the Department of Radiation Oncology. The patient is planned for an annual follow-up with PET-CECT imaging and clinical review.

## Discussion

Malignant nodular hidradenomas are a group of aggressive and poorly differentiated eccrine sweat gland tumours that invade surrounding tissue and lymphovascular structures. It has equal male-female distribution and a high local recurrence rate, usually, within the first two years from diagnosis [1]. While usually involving the head and neck region and the anterior truncal surface, eccrine tumours of the extremities are extremely rare. The treatment of choice is wide local excision and the role of adjuvant therapy is controversial [2].

Clinically, hidradenomas present in an asymptomatic indolent manner as swelling with slow progression, with drastic unheralded malignant transformation with lymphatic spread [3] and metastases [4]. The patient usually presents with localized discomfort, pain, or ulceration with bleeding. It can be mistaken for cutaneous tuberculosis and dermatofibrosarcoma protuberans [5]. Hidradenomas frequently present themselves in the areas of the head and neck; the incidence of extremity hidradenomas is rare [6]. If lymphatic involvement is present, visceral metastasis usually follows in 28% of patients with hidradenoma [7].

The histopathology and immunohistochemistry of malignant nodular hidradenoma appear similar to ductal adenocarcinoma of breast and salivary gland tumours [1].

Histology of the primary neoplasm usually shows nodular dermal proliferation composed of poorly differentiated clusters of epithelioid cells with the pseudopapillary arrangement. For diagnosing a malignant hidradenoma, criteria typically include deep extension with an infiltrative pattern, nuclear pleomorphism, necrosis, increased mitoses (>4 per HPF), and lympho-vasculoneural invasion [8]. However, the absence of any of the above criteria does not preclude the diagnosis of malignant hidradenoma.

There are few existing immunohistochemical studies due to the rarity of the disease and due to misdiagnoses. Some markers that were identified were p53, Ki-67 protein (as a proliferation marker), MIB-1 index (a marker for adnexal neoplasms), PHH3 marker (specific for mitosis), epithelial membrane antigen, CK 5/6, BCL - 1/2 and EGFR (cellular signalling pathways). In the study done by Ko et al., all tumour specimens included in the study showed positivity for keratin AE1/3 and cytokeratin 5/6 [9].

Radiologically, magnetic resonance imaging shows soft tissue mass in subcutaneous layers with well-delineated margins and heterogeneous appearance - lobulated, cystic, or solid. T1 weighted images can reveal low to intermediate signal intensity lesions with markedly isointense areas. On T2 weighted images, the lesion shows intermediate to high signal compared to muscle with heterogeneity. The variation is most likely due to the variations in adnexal secretions, blood, and fatty tissues in the cystic fluid. PET CT usually shows hypermetabolic activity. The utility of PET-CT has been demonstrated in following up on post-treatment patients by monitoring the metabolic response. PET CT has been previously used for patients with abnormal skin neoplasms, but with a caveat that dermal metastases of internal malignancies might be a false positive, albeit low in incidence [10,11].

It is primarily treated by surgical excision with a tumour-free margin of 2 cm [2] and the efficiency of adjuvant therapy for positive margins - systemic chemotherapy and radiotherapy is not well established. Prophylactic lymph nodal dissection has not been proven to improve the disease-free interval [1]. It was found that the disease recurred in 50-60% of patients either locoregionally or as metastases despite complete wide local excision with clear margins. The five-year post-surgical survival rate for malignant hidradenoma is reported to be lesser than 30% [12]. Moh’s micrographic surgery is a treatment modality with promising results with no recurrence, metastasis, or disease-related mortality [13,14].

Systemic chemotherapy regimens based on fluorouracil, cisplatin, and doxorubicin have not shown any significant therapeutic benefit. In a few cases, malignant eccrine tumours with receptors positive for estradiol showed response to tamoxifen therapy with complete resolution of lymph node spread and full pain relief from bone metastasis. The pain relief from bone metastasis was present for a period of nearly three years [15,16].

Radiotherapy as an adjuvant modality of treatment has been established in the 1990s for head and neck malignant hidradenoma with positive margins. Adjuvant modalities of treatment acquire more importance as the tumour has a 10-50% chance of recurrence despite aggressive surgical treatment [17]. Histopathological factors that herald the necessity of adjuvant radiotherapy include anaplastic tissue architecture, lympho-neurovascular invasion, positive margins, extracapsular lymphatic extension, and nodal sterilization [18,19]. Concurrent chemoradiation has no effect on local disease control or overall survival [20].

Additionally, 71.6 Gy high dose irradiation with 57.6Gy to the tumour bed and 14Gy boost to the primary lesion using high energy photon with or without electron boost provided a disease-free period of 35 months after treatment completion. Hyperfractionation is recommended to prevent adverse reactions, especially in the head and neck area [19].

Another modality of treatment is electrochemotherapy involving local or intravenous antineoplastic agent instillation, usually bleomycin or cisplatin with electroporation of the tumour, thus exposing the tumour cells to cytotoxic drugs. It is a well-tolerated treatment modality suitable for cutaneous and subcutaneous tumours with minimal side effects, with the maintenance of cosmesis and function of surrounding tissues, but warrants a larger sample size and widespread usage before deciding upon it as a standard of care [20].

As a rule, the principles of treatment of melanoma and other skin cancers have been applied to the treatment of adnexal skin tumours. Thus, it warrants precise and careful surgical excision, regular follow-up, and investigations into the utility of sentinel lymph node biopsy.

Due to the pandemic and the age of the patient, we had opted for a wait-and-watch approach rather than the usual modalities of treatment. The patient is under expectant management with our department.

## Conclusions

This case report is to highlight the need to be aware of and recognize a seemingly harmless swelling before it manifests in its natural course of aggression. It also suggests the need to evaluate such rare cases on a per-patient and per-situation basis and decide on treatment accordingly

Robust follow-up policies are required for enabling wait and wat,ch protocols, and in malignant nodular hidradenoma, it could be scheduled as six-month follow-ups imaging reviews for the first three years and annually thereafter, with clinical examination and assessment every three months for the first two years, every six months for the next three years and annually thereafter.
